# Supplementary material for: Suicide-Gene-Modified Extracellular Vesicles of Human Primary Uveal Melanoma in Future Therapies
Source: Int J Mol Sci. 2023 Aug 19;24(16):12957. doi: 10.3390/ijms241612957 (PMC10454466; doi:10.3390/ijms241612957)
Supplement: Supplementary file 1 [file ijms-24-12957-s001.zip › Supplementary Table S1_editable.pdf]

Supplementary Table S1.

Antibody	Species/Isotype	MW (kDa)	Dilutions WB	Dilute in	Company №
**CD9 (Ts9)**	Mouse / IgG1	24	1ug/ml	5% MILK	Invitrogen #10626D
**CD63 (Ts63)**	Mouse / IgG1	40-50	2ug/ml	5% MILK	Invitrogen #10628D
**CD81 (M38)**	Mouse / IgG1	25	2ug/ml	5% MILK	Invitrogen #10630D
**ITG α5/CD49e**	Mouse / IgG2a	150	1:500	5%MILK	BD Biosciences #610633
**Integrin β1**	Rabbit IgG	115/135	1:1000	5%MILK	CST #4706S
**Anti-β-Actin**	Mouse/ IgG	42	1:2000-1:4000	0.1% TBST	Sigma-Aldrich # A1978
**Alexa Fluor 680**	Secondary Antibody, Goat anti-Mouse IgG (H+L)		1:5,000-1:20,000 opt. 1:10,000	0.1% TBST	Invitrogen #A-21058
**Anti-rabbit IgG, HRP-linked Antibody**	Goat polyclonal Secondary ab. to Rabbit IgG H&L (HRP)		1:1000-1:3000	5% MILK	CST #7074S
**Anti-mouse IgG, HRP-linked Antibody**	Secondary ab. to mouse IgG H&L (HRP)		1:1000-1:3000	1% BSA 0,1% TBST	CST #7076S
