# Supplementary figures and images for: Suicide-Gene-Modified Extracellular Vesicles of Human Primary Uveal Melanoma in Future Therapies
Source: Int J Mol Sci. 2023 Aug 19;24(16):12957. doi: 10.3390/ijms241612957 (PMC10454466; doi:10.3390/ijms241612957)

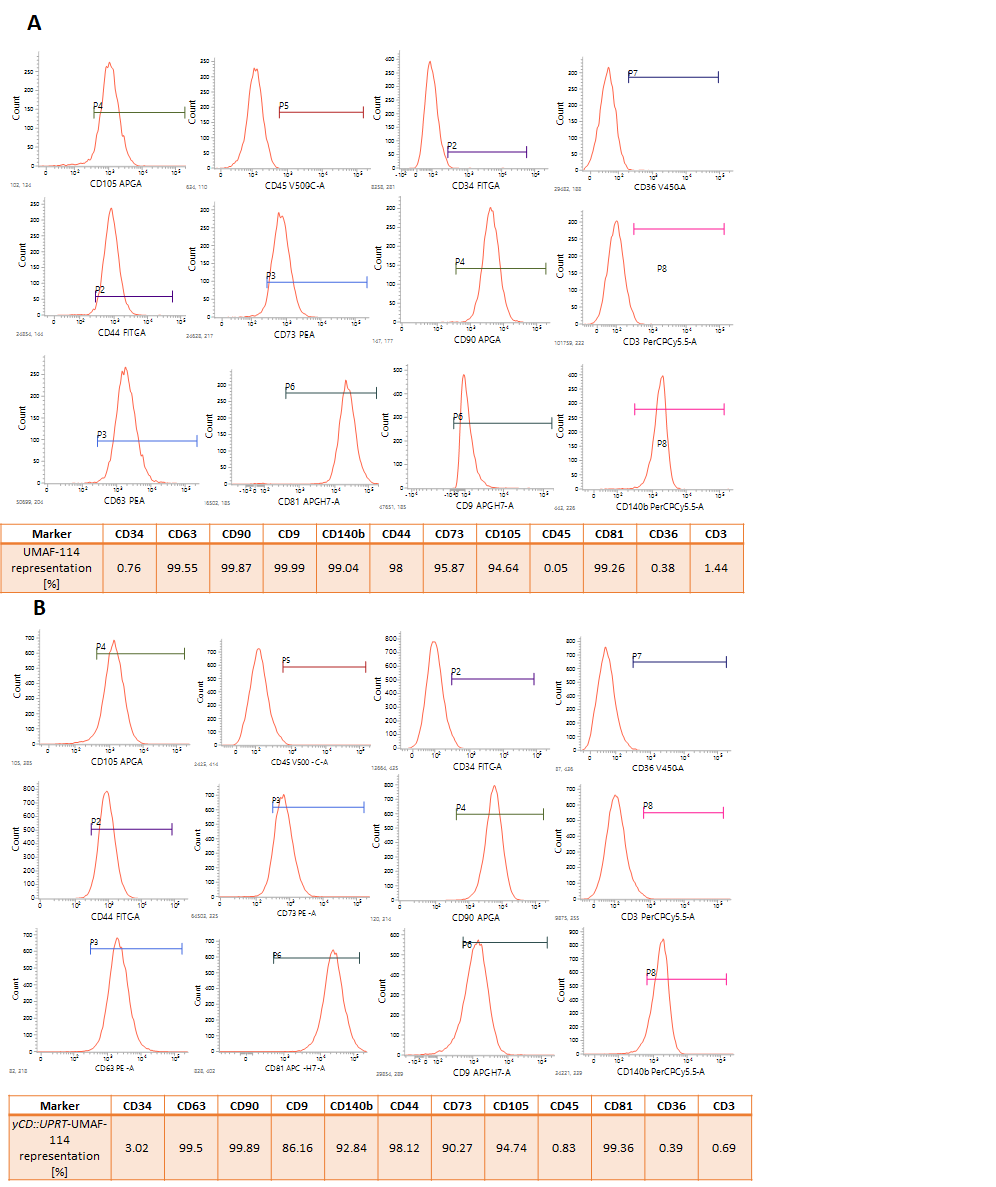

Supplement: Supplementary file 1 [file ijms-24-12957-s001.zip › Supplement Figure S1.png]
